# Single-Cell Transcriptomics of Glioblastoma Reveals a Unique Tumor Microenvironment and Potential Immunotherapeutic Target Against Tumor-Associated Macrophage

**DOI:** 10.3389/fonc.2021.710695

**Published:** 2021-08-09

**Authors:** Xiaoteng Cui, Qixue Wang, Junhu Zhou, Yunfei Wang, Can Xu, Fei Tong, Hongjun Wang, Chunsheng Kang

**Affiliations:** ^1^Lab of Neuro-oncology, Tianjin Neurological Institute, Key Laboratory of Post-Neuroinjury Neuro-repair and Regeneration in Central Nervous System, Department of Neurosurgery, Tianjin Medical University General Hospital, Tianjin, China; ^2^Department of Neurosurgery, Affiliated Hospital of Hebei University, Baoding, China; ^3^Department of Neurosurgery, The Second Affiliated Hospital of Harbin Medical University, Harbin, China

**Keywords:** immune landscape, glioblastoma, single-cell RNA sequencing, macrophage polarization, SPI1

## Abstract

**Background:**

The main immune cells in GBM are tumor-associated macrophages (TAMs). Thus far, the studies investigating the activation status of TAM in GBM are mainly limited to bulk RNA analyses of individual tumor biopsies. The activation states and transcriptional signatures of TAMs in GBM remain poorly characterized.

**Methods:**

We comprehensively analyzed single-cell RNA-sequencing data, covering a total of 16,201 cells, to clarify the relative proportions of the immune cells infiltrating GBMs. The origin and TAM states in GBM were characterized using the expression profiles of differential marker genes. The vital transcription factors were examined by SCENIC analysis. By comparing the variable gene expression patterns in different clusters and cell types, we identified components and characteristics of TAMs unique to each GBM subtype. Meanwhile, we interrogated the correlation between SPI1 expression and macrophage infiltration in the TCGA-GBM dataset.

**Results:**

The expression patterns of TMEM119 and MHC-II can be utilized to distinguish the origin and activation states of TAMs. In TCGA-Mixed tumors, almost all TAMs were bone marrow-derived macrophages. The TAMs in TCGA-proneural tumors were characterized by primed microglia. A different composition was observed in TCGA-classical tumors, which were infiltrated by repressed microglia. Our results further identified SPI1 as a crucial regulon and potential immunotherapeutic target important for TAM maturation and polarization in GBM.

**Conclusions:**

We describe the immune landscape of human GBM at a single-cell level and define a novel categorization scheme for TAMs in GBM. The immunotherapy against SPI1 would reprogram the immune environment of GBM and enhance the treatment effect of conventional chemotherapy drugs.

## Introduction

Glioblastoma (GBM), which comprises grade IV gliomas, is a notoriously malignant brain tumor with high inter- and intratumoral heterogeneity (1, 2). Based on integrated genomic analysis from both bulk RNA sequencing and single-cell RNA sequencing (scRNA-seq) of GBM tumors, four distinct molecular subtypes have been described. These subtypes (referred to as classical, neural, proneural, and mesenchymal) were defined based on unique characteristics regarding DNA copy number variations, somatic mutations, and transcriptional profiles (3–6). The subtypes have also been identified in different regions of the same tumor, suggesting that the original tumor included a combination of various cells with distinct transcriptomic features (7, 8). Genetic mutations, epigenetic alterations, and differing tumor microenvironments alter the cellular character of GBM and drive its heterogeneity, and are among the primary reasons for the dismal prognosis and inevitable therapeutic resistance of this cancer (9, 10). The standard treatment for GBM is extensive surgery resection, followed by adjuvant radiotherapy and chemotherapy with temozolomide. However, the clinical efficacy of this regimen is still limited, and the median survival of GBM patients is less than 1½ years (1, 11, 12). Recently, various immunotherapies directed towards different targets, especially the programmed cell death protein 1 (PD-1)/PD-1 ligand (PD-L1) pathway, have been revealed as favorable strategies to treat several tumor types, such as melanoma and non-small-cell lung cancer (13, 14). However, contrary to expectations, the treatment of GBM with a PD-1 inhibitor did not yield a satisfactory response (15, 16). As the nature of the immune cells involved in GBM correspond with treatment efficacy, it is critical to illustrate the GBM immune environment by clarifying the orientation and state of tumor-infiltrated immune cells and characterizing their transcriptomic features.

The tumor microenvironment contains complex cellular components, including non-neoplastic cells such as myeloid cells and lymphocytes (17, 18). In GBM, the majority of immune cells are tumor-associated macrophages (TAMs), which comprise 30–40% of the cell mass, whereas T cells account for less than 0.23% of the total cell count (19, 20). TAMs in GBM consist of two different cell populations: resident microglia, which arise during brain development (21), and bone marrow–derived macrophages that infiltrate the tumor *via* blood vessels (22). However, the activation states and transcriptional features of immune cells in GBM remain unclear.

scRNA-seq has been widely used to comprehensively characterize gene expression at the level of individual cells, and it has been employed to analyze intratumoral heterogeneity in glioma (23). In the present study, we analyzed data from scRNA-seq that was performed using tissue from nine glioblastomas, covering 16,201 cells in total. These data were integrated to depict the immune landscape of glioblastoma at the single-cell level. Primed and repressed TAM states were defined based on the expression profiles of key genes. By comparing variable gene expression and regulons in different clusters and cell types, we not only identified components and characteristics unique to each GBM subtype but also discovered a potential immunotherapeutic target for GBM, Spleen Focus Forming Virus Proviral Integration Oncogene (*SPI1*), which is expressed in TAM and essential for macrophage maturation and polarization, and correlated with tumor grades and poor prognosis.

## Materials and Methods

### Acquisition of scRNA-Seq Datasets

Normalized expression matrices processed as Transcripts Per Kilobase of exon model per Million mapped reads (TPM) from scRNA-seq performed using a 10 × genomics platform were downloaded from the Gene Expression Omnibus (GEO) (https://www.ncbi.nlm.nih.gov/geo/) database. The sequencing analyses were conducted on samples from nine fresh GBM tumors from adult patients in Massachusetts General Hospital (MGH) (GSE131928) (4). A total of 16,201 cells were acquired. A seurat file was created by importing the expression matrix data into the version 3.6.2 R studio program and saving the data in RDS format.

### Dimensionality Reduction and Unsupervised Clustering

The data were normalized using the global-scaling method “LogNormalize” and preprocessing the data by linear conversion. To detect the most variable genes used for dimensionality reduction, we employed the “FindVariableFeatures” function in R studio. Next, we utilized PCA to reduce the dimensional linearity based on the variable genes. JackStraw plots and Elbow plots were used to decide the number of dimensionalities. Finally, graph-based unsupervised clustering was conducted and visualized using a non linear t-SNE plot, with a resolution of 0.4, defined by the functions of “FindNeighbors” and “FindClusters”. All the operations were processed by using the seurat R package in R studio.

### Identification of Cell Types and Marker Genes in Different Clusters

To identify the cell types, we first analyzed the Spearman correlation coefficient between the transcriptomes of each cell across all nine tumors in our dataset and each cell type-specific gene expression profile in the HPCA reference set using SingleR package. Then, the expression levels of distinct cell type-specific marker genes based on the CellMarker databases (24) were utilized to further confirm the cell types. Markers for macrophage were *C1QA*, *C1QB*, *C1QC*, *TYROBP*, and *CD68*. For T lymphocyte, the markers were *CD3G*, *GZMH*, *IL2RB*, *PRF1*, and *ICOS*. *TMEM119* was employed to distinguish the microglia from TAM clusters. The relatively positive expressions of *MHC-II molecules* in TAMs were considered as distinct features of primed state. The cell type information was visualized by t-SNE plot in R studio.

The marker genes of distinct clusters were identified using the “FindAllMarkers” function of the seurat package and visualized as a heat map, violin plots, and feature plots using the ggplot2 package in R studio. The positive percentage of the cells where the gene is defined as the marker is at least 25%.

### Identification of Gene Regulatory Network and Specific Transcription Factors

The gene regulatory network and specific transcription factors were identified by employing the Single Cell Regulatory Network Inference and Clustering (SCENIC) tool (25) using default parameters. The SCENIC analysis was run as described on the cells that passed the filtering using the 20,000 motifs database for RcisTarget and GRNboost R packages. The regulated genes and AUCell score of each transcription factor were calculated to estimate the specificity to each cell type. The AUCell scores were visualized by t-SNE plot and heat map. The top three regulons in each cell type were acquired by calculating the RSS. The relationships among transcription factors were shown in heat map by connection specificity index (CSI).

### KEGG Pathway Analysis

DAVID (https://david.ncifcrf.gov/) is an open website providing a comprehensive set of functional annotation tools to extrapolate biological meaning from an extensive list of genes. Using DAVID, we identified the main cellular physiological activities reflected by the top 500 differentially expressed genes of each cluster. The KEGG pathways were displayed as a bubble plot by the ggplot2 package in R studio.

### Gene Expression Analysis

The expression data of SPI1 and the clinical information of glioma samples were obtained from TCGA, CGGA (26), and Rembrandt databases. The data were sorted into three subgroups according to the WHO grade and used to calculate the mean and 95% confidence interval (CI) of SPI1 expression. The results were visualized as scatter plots.

### Correlation Analysis

We utilized TIMER2.0 (Tumor Immune Estimation Resource version 2.0) online web (http://timer.cistrome.org/) (27) to analyze the correlation between the expression of SPI1 and other genes. We input “SPI1” in the “Interested Gene” search box of the “Cene_Corr” module in TIMER2.0 and “C1QA”, “C1QB”, “C1QC”, “SRGN”, “TYROBP”, “GAP43”, “GPM6B”, “SEC61G”, “PTN”, “CX3CR1”, “CSF1R”, and “IRF8” in the “Gene Expression” box, and obtained the correlation in all tumor types. We picked up the GBM results and saved the scatter plots with rho and p value.

### Tumor Purity and Survival Prognosis Analysis

The tumor purity, macrophage infiltration, and survival prognosis analysis were performed as previously described (28).

## Results

### scRNA Sequencing Reveals the Unique Immune Landscape of Human GBM at a Single-Cell Level

**Figure 1:**
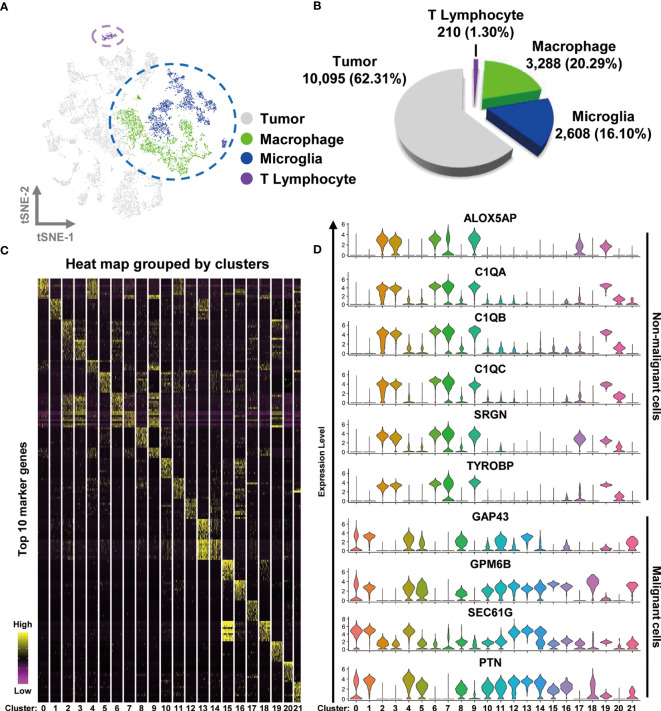
Integrated scRNA-seq analysis from human GBM biopsies. **(A)** A total of 16,201 cells from nine datasets of fresh GBM tissues are visualized by the t-SNE plot. Distinct cell types are represented as follows: light gray indicates tumor cells, green indicates macrophages, cyan indicates microglia, and purple indicates T lymphocytes. **(B)** A pie chart illustrates the proportions of each cell type. **(C)** The expression profiles of the top 10 marker genes of each cluster are displayed as a heat map. **(D)** Distributions of malignant and non malignant cell marker genes are shown as violin plots.

To examine the immune microenvironment of GBM, we obtained 10 × Genomics scRNA sequencing datasets derived from fresh GBM tissues that spanned nine patients, accessed from the Gene Expression Omnibus (GEO) database (accession number GSE131928) (4). A total of nine datasets were integrated and comprehensively analyzed to assess GBM heterogeneity and determine the nature of various associated immune microenvironments. We used t-distributed stochastic neighbor embedding (t-SNE), the unsupervised non linear dimensionality reduction algorithm, to differentiate cell types based on their relative gene expression values. Overall, single-cell transcriptomes for a total of 16,201 cells were retained after initial quality controls and were differentiated into 22 different clusters (cluster 0~-21), visualized as bidimensional t-SNE maps (**Figure S1A**). The distribution of each dataset is displayed in **Figure S1B**. The clusters were sorted into four main cell types by comparing their gene expression characteristics for tumor (light gray), macrophages (green), microglia (blue), and T lymphocyte (purple) (**Figure 1A**). Surprisingly, the number of malignant cells (tumor) accounted for 62.31% (10,095 cells) of the cell population, suggesting that numerous tumor-associated non-malignant cells are a prominent feature of the GBM microenvironment. We observed that TAMs (macrophage and microglia) comprised 36.39% of the tumor tissue cells (the numbers of macrophage and microglia were 3,288 and 2,608 cells, respectively), and the percentage of T cells was only 1.30% (210 cells) of the total cell count (**Figure 1B**). The percentages of cell types in each sample are shown in **Figure S2**.

Heat mapping was performed to examine the expression levels of the top 10 genes in the different clusters. As shown in **Figure 1C** and **Table S1**, each cluster displayed distinct gene expression features. The clusters in the TAM group (particularly clusters 2, 3, 6, 7, 9, and 19) shared a number of similar marker genes, such as complement c1q C chain (*C1QC*), complement c1q B chain (*C1QB*), *Metallothionein 1G* (*MT1G)*, serglycin (*SRGN*), c-c motif chemokine ligand 3 like 3 (*CCL3L3*), major histocompatibility complex class II DR beta 5 (*HLA-DRB5*), and s100 calcium binding protein A8 (*S100A8*). By analyzing the expression of different genes, we observed that TAMs could be identified by the expression of Arachidonate 5-Lipoxygenase Activating Protein (*ALOX5AP*), complement c1q A chain (*C1QA*), *C1QB*, *C1QC*, *SRGN*, and TYRO protein tyrosine kinase binding protein (*TYROBP*), whereas malignant cells expressed the marker genes growth-associated protein 43 (*GAP43*), glycoprotein M6B (*GPM6B*), SEC61 translocon subunit gamma (*SEC61G*), and pleiotrophin (*PTN*) (**Figure 1D**). The non malignant cells were differentiated into T cells and TAMs by the expression of the genes CD3 gamma chain (*CD3G*), granzyme H (*GZMH*), interleukin 2 receptor subunit beta (*IL2RB*), perforin 1 (*PRF1*), and inducible T cell costimulator (*ICOS*) (**Figure S1C**). The non malignant and malignant cells were also easily separated in bidimensional PC maps, which rely on linear principal component analysis (PCA) to reduce dimensionality (**Figure S1D**).

Altogether, our data suggest that TAMs are the most common immune cell type in GBM, comprising over half of the tumor cell population.

### Primed and Repressed States of Macrophages/Microglia Characterize the Immune Microenvironment in GBM

To comprehensively characterize the signatures of TAMs in GBM, we subclustered and re analyzed the cells in the macrophage and microglia group. In total, 5,896 cells were sorted and hierarchically sorted into 14 clusters (**Figure S3A**). *CD68* has been widely recognized as a pan-macrophage marker, and we therefore used feature and violin plots to visualize the expression and distribution of *CD68* in these clusters. As shown in **Figures S3B, C**, *CD68* was highly expressed in almost every cluster, except for cluster 12, which may be tumor cells misdefined as TAMs according to the gene signature above. Then, we further analyzed the *CD68*-positive clusters, which we considered to be TAMs. The macrophage compartment in GBM contains both brain-resident microglia and bone marrow–derived macrophages. *TMEM119* is a reliable microglia marker that discriminates inherent and extrinsic macrophages in the brain (29). Using the violin plot of *TMEM119* expression, we separated the macrophage clusters into two groups. The *TMEM119*-positve clusters (clusters 4, 5, 6, 7, 10, and 11) were identified as resident microglia, and the *TMEM119*-negative clusters (clusters 0, 1, 2, 3, 8, 9, and 13) were defined as bone marrow–derived macrophages (**Figure S3D**). By examining the marker genes defining the *TMEM119*-positive clusters, we selected cytoplasmic fmr1 interacting protein 1 (*CYFIP1*), ectonucleoside triphosphate diphosphohydrolase 1 (*ENTPD1*), and vav guanine nucleotide exchange factor 1 (*VAV1*) as possible microglia marker genes (**Figure S3D**). MHCII is a glycoprotein present on specialized antigen-presenting cells, including macrophages. The elevated expression of MHCII indicates that macrophages have transitioned to a primed state, in which they have acquired the ability to present tumor-specific antigens (30). Macrophages otherwise maintain a repressed state. We therefore examined the expression of *MHCII* by assessing the levels of the human genes, which were HLA-DQ alpha 2 (*HLA-DQA2*) and HLA-DQ beta 2 (*HLA-DQB2*). As shown in **Figure S2E**, HLA-positive expression was confined to clusters 1, 2, 4 5, 6, 8, and 9. Together with their expression of *TMEM119*, the cells were subdivided into four distinct groups as follows: TMEM119^+^-HLA^+^ cells (clusters 4, 5, and 6), TMEM119^+^-HLA^−^ cells (clusters 7, 10, and 11), TMEM119^−^-HLA^+^ cells (clusters 1, 2, 8, and 9), and TMEM119^—^HLA^−^ cells (clusters 0, 3, and 13) (**Figure S3F**).

Next, we wished to determine the precise nature and activation status of TAMs in GBM. In our analysis of the distinctive gene expression profiles of microglia, we found that the cell population of cluster 11 charactered by matrix metallopeptidase 9 (MMP9), MMP25, and periostin (POSTN), which are associated with cell migration, should be more likely microglia with naïve status as an immunological defender in the brain, which has migration and chemotaxis ability. We defined cells with this expressions profile as “microglia: unactivated”. Similarly, the expression of cytokine-like 1 (CYTL1) and early growth response 3 (EGR3) were used to define primed microglia. Interferon-induced protein with tetratricopeptide repeats 3 (IFIT3) and interferon alpha inducible protein 27 (IFI27) distinguished microglia in the repressed state. The gene profile analysis of macrophages suggested that cluster 8 represented priming macrophages, owing to the expression of cell cycle-associated genes [aurora kinase B (AURKB), cell division cycle associated 3 (CDCA3), and assembly factor for spindle microtubules (ASPM)]. HLA-positive macrophages could be classified as primed, and HLA-negative macrophages (with the exception of cluster 8) were categorized as repressed, due to the expression of MT1G and ankyrin repeat domain 28 (ANKRD28) (**Figures S4A, B**).

**Figure 2:**
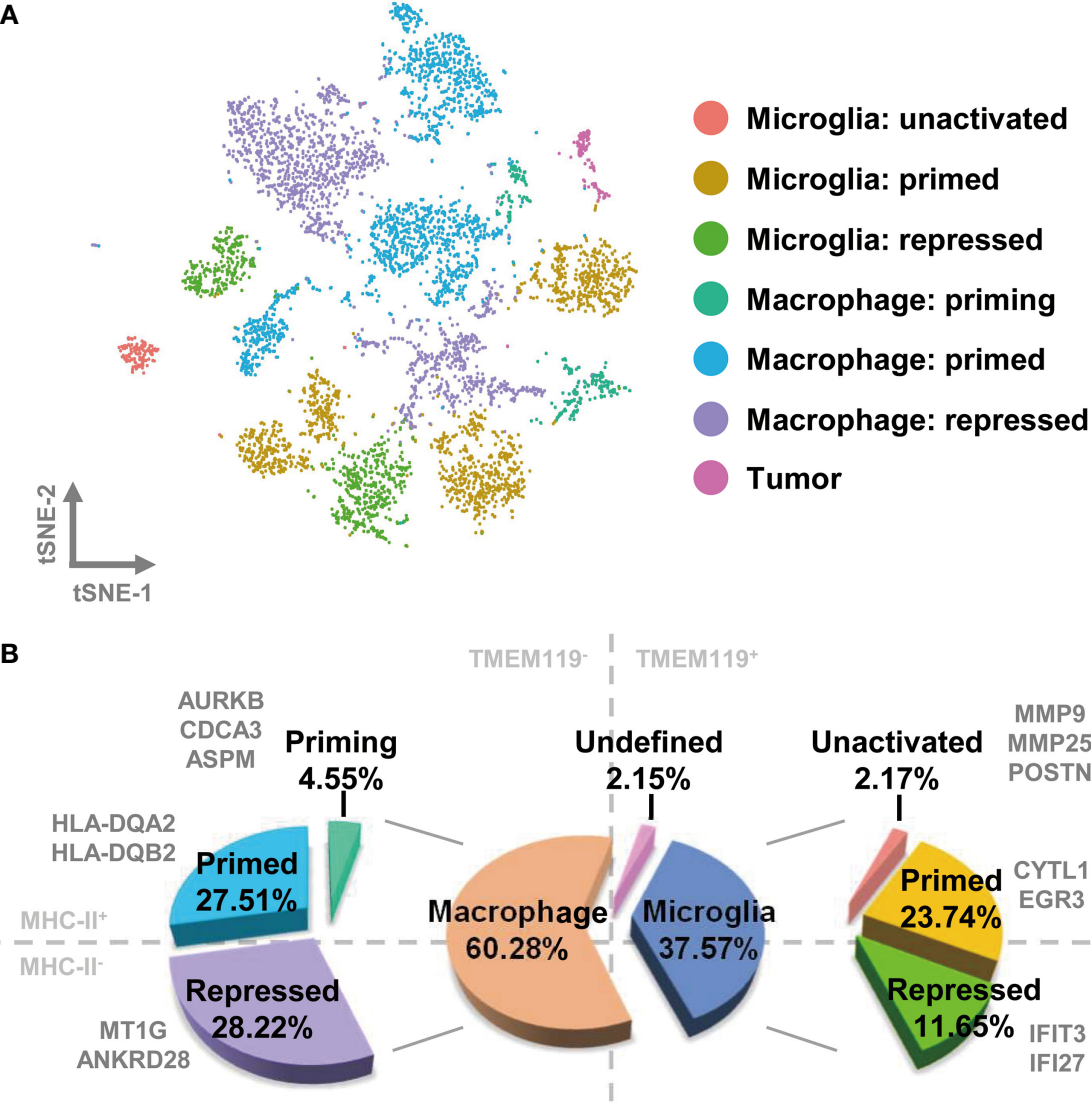
TAMs in GBM comprise different activation states of bone marrow–derived macrophages and brain-resident microglia. **(A)** Each cell type is visualized as a t-SNE plot. **(B)** The relative proportions of macrophages and microglia are 60.28% and 37.57%, respectively. The ratio of detailed TAM states is shown in the pie chart.

With all of the above characteristics in mind, we redefined the original 14 clusters as seven distinct subsets by comparing the expression of *TMEM119* and *MHCII* with the expression profiles of different clusters (**Figures 2A** and **S4C**). TAMs in GBM consist primarily of bone marrow–derived macrophages, accounting for 60.28% of the cell population, of which 4.55% are undergoing priming, 27.51% are in the primed state, and 28.22% are repressed. Of the remaining cell population, 37.57% consists of resident microglia in unactivated (2.17%), primed (23.74%), and repressed states (11.65%) (**Figure 2B**).

### The Orientation and Status of TAM in Different Subtypes of GBM Are Distinctive

**Figure 3:**
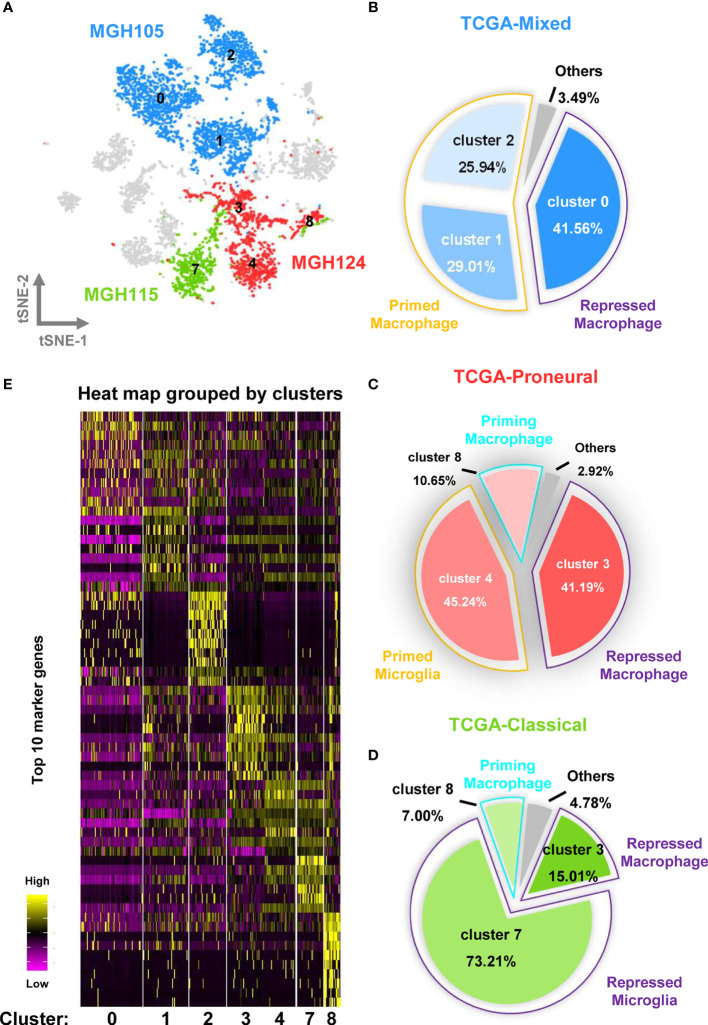
Different molecular subtypes of GBM have distinct TAM composition and characteristics. **(A)** The cell distribution of MGH105, MGH115, and MGH124 are shown by the t-SNE plot. **(B)** TAMs in the TCGA-mixed tumor comprise repressed macrophages, primed macrophages, and others. **(C)** TAMs in the TCGA-proneural tumor comprise repressed macrophage, primed microglia, priming macrophages, and others. **(D)** TAMs in the TCGA-classical tumor contain repressed macrophages, repressed microglia, priming macrophages, and others. **(E)** The expression profiles of the top 10 marker genes of each cluster are displayed as a heat map.

To investigate the relationship between the infiltrated TAMs and different GBM subtypes, we assessed the distribution of the cells covered by the nine tumor datasets and selected the MGH105 (40.28%), MGH124 (18.00%), and MGH115 (9.94%) datasets for analysis, as together they covered over half of the total cell number and seven different clusters (**Figures 3A** and **S5**). Fittingly, we found that these datasets represented diverse tumor subtypes previously defined by TCGA (MGH105 was classified as “mixed”, MGH115 as “classical”, and MGH124 as “proneural”) (4), and we therefore examined the characteristics of these distinct GBM subtypes using these three datasets. In the TCGA-mixed tumor, almost all TAMs in the GBM were bone marrow–derived macrophages, with primed state cells occupying 54.95% of the total number (clusters 1 and 2) and repressed state cells accounting for 41.56% (cluster 0) (**Figure 3B**). The TAMs in the TCGA-proneural tumor were characterized by primed microglia (cluster 4; 45.24%), repressed macrophages (cluster 3; 41.19%), and priming macrophages (cluster 8; 10.65%) (**Figure 3C**). A different composition was observed in the TCGA-classical tumor, with repressed microglia (cluster 7) comprising 73.21% of the total cell number and other TAM subtypes, such as repressed macrophages (cluster 3; 15.01%) and priming macrophages (cluster 8; 7.00%), making up the rest of the cell proportion (**Figure 3D**).

**Figure 4:**
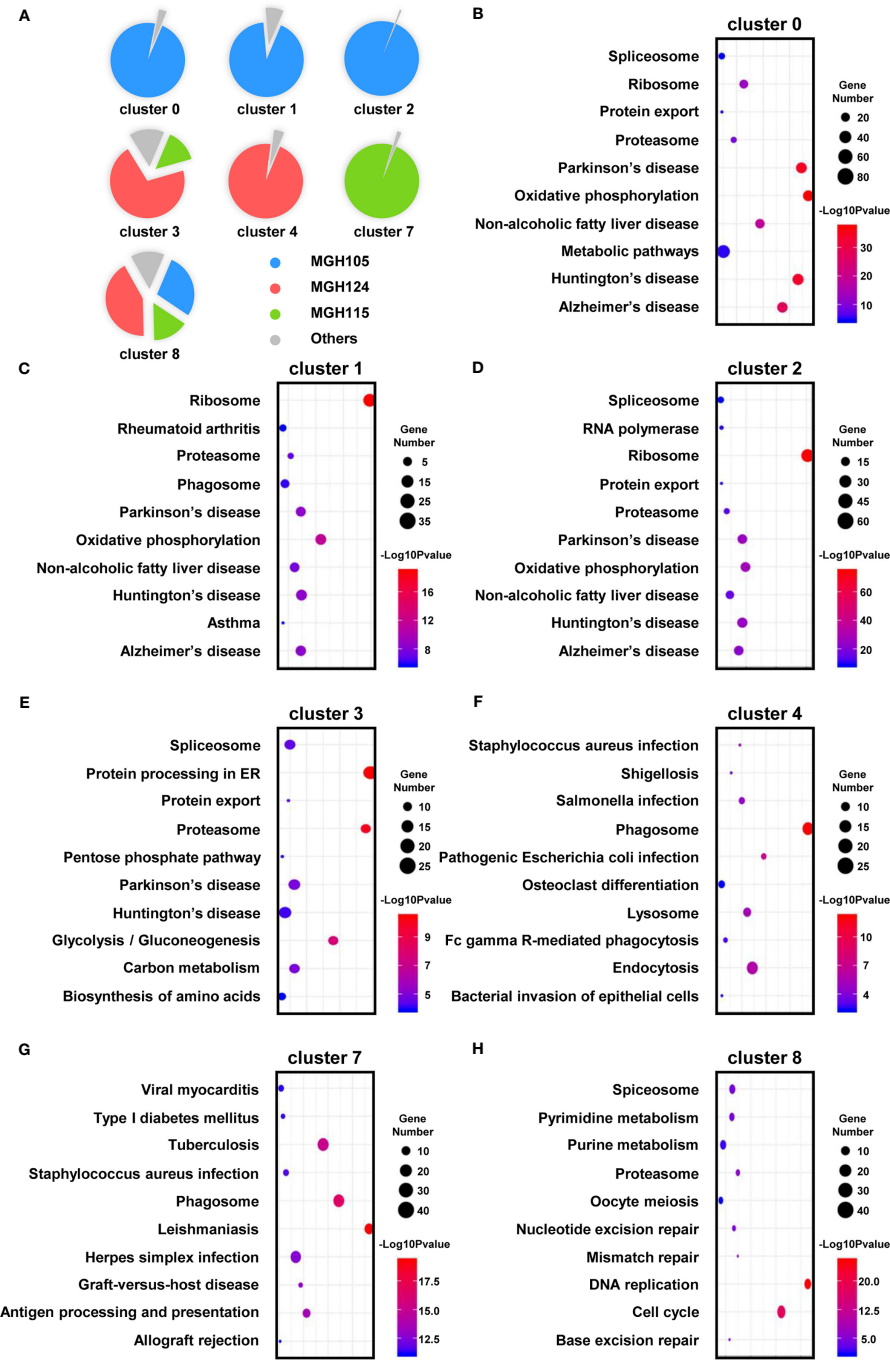
The enriched cellular physiological activities of TAMs differ from each other. **(A)** Each cluster contains at least three of the nine tissues. **(B–H)** The enriched KEGG pathways of each cluster are shown in bubble charts.

Heat map analysis showed that macrophages/microglia in the same activation state possessed distinct gene expression profiles in the different subtypes of GBM (**Figure 3E** and **Table S2**). Each of the clusters included cells present in at least three of the nine datasets (**Figure 4A**). To further clarify the main cellular physiological activities of TAMs, the top 500 differentially expressed genes in each cluster were identified and evaluated by KEGG pathway analysis. As shown in **Figures 4B, E**, “oxidative phosphorylation” was one of the most repressed macrophages from the TCGA-mixed tumor (cluster 0), but “protein processing in ER” was the most enriched set of pathways in the same macrophages from the TCGA-proneural and classical tumors (cluster 3). Primed state macrophages in the TCGA-mixed tumor (clusters 1 and 2) were enriched in the “ribosome” pathway (**Figures 4C, D**). Cluster 4, which represents primed microglia that make up the highest percentage of the cells in the TCGA-proneural tumor, was characterized by the KEGG enrichment term “phagosome” (**Figure 4F**). The pathways associated with “leishmaniasis” were enriched prominently in repressed microglia from the TCGA-classical tumor (cluster 7) (**Figure 4G**). The only TAM cluster present in all three subtypes of GBM was priming macrophages (cluster 8), which were enriched for pathways involved in “DNA replication” and the “cell cycle” (**Figure 4H**).

### SPI1 Is a Crucial Regulon for TAM Maturation and Polarization and Correlated With Poor Prognosis in GBM

**Figure 5:**
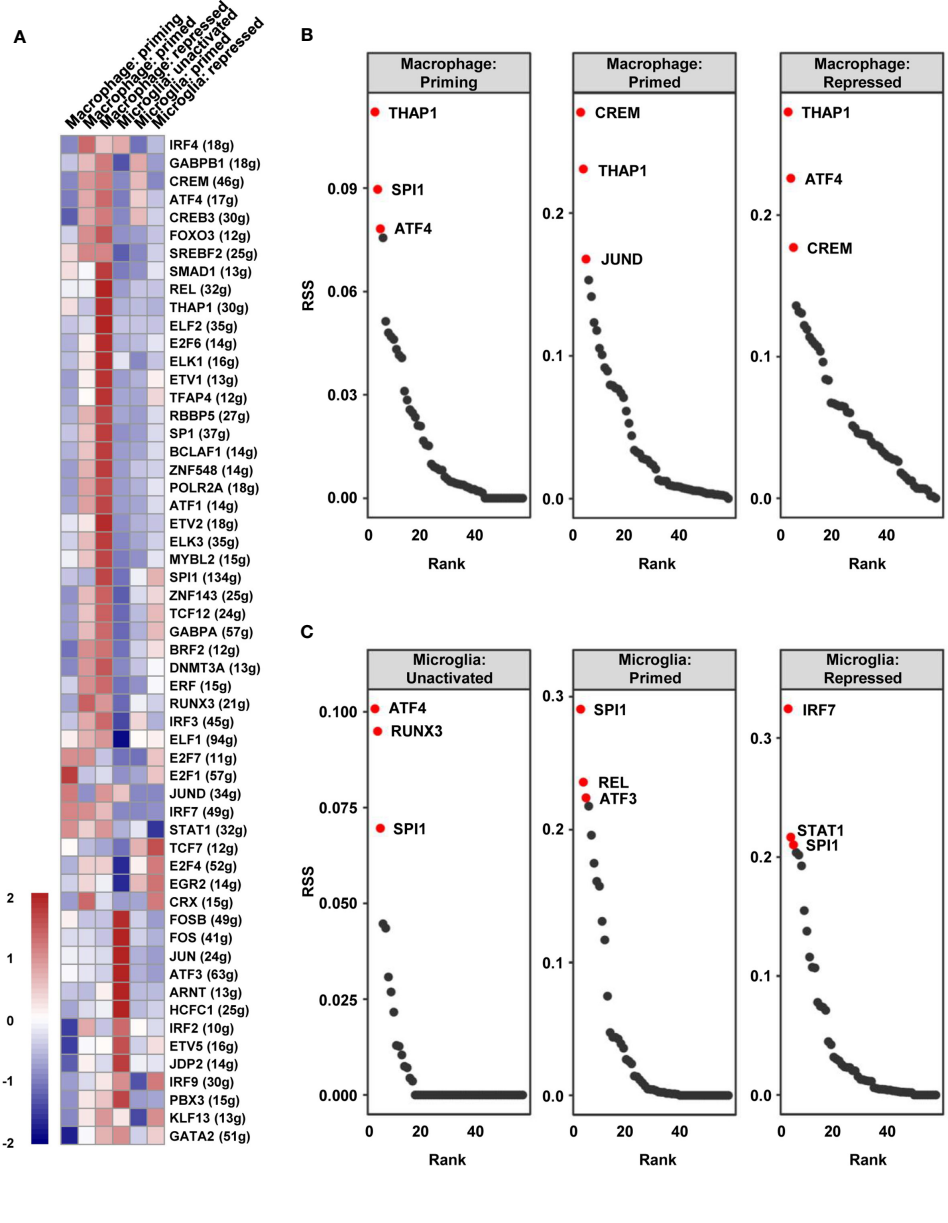
The vital regulons in each cell type of TAMs. **(A)** The RAS of every regulon in different cell types was visualized as a heat map. **(B, C)** The ordered regulons based on the RSS were shown as sequence diagrams.

To identify the distinct key transcription factors of different states of macrophages/microglia and compare the regulon differences, Single-Cell rEgulatory Network Inference and Clustering (SCENIC) was employed to draw the gene regulatory network. Based on the calculated regulon activity score (RAS) of transcription factors, we could identify the differences in the activities of regulons among the cell types (**Figure 5A**). The relevance among the regulons was evaluated by connection specificity index (CSI) and visualized in **Figure S6**. Some regulons, such as E2F transcription factor 7 (E2F7) and Cone-Rod Homeobox (CRX), had elevated activities in primed macrophage but decreased activities in repressed macrophage. Meanwhile, some others including SMAD Family Member 1 (SMAD1) and JunD proto-oncogene (JUND) showed low activities in primed but a high activities in repressed state of macrophages. Similarly, high activities of cAMP-responsive element modulator (CREM) and interferon regulatory factor 3 (IRF3) were calculated in primed microglia, but Transcription Factor 7 (TCF7) and interferon regulatory factor 9 (IRF9) in repressed microglia. By calculating the regulon specificity score (RSS), we examined the crucial regulons of each cell type and visualized the top three regulons in sequence diagrams and t-SNE plots (**Figures 5B, C** and **S7A–I**). Surprisingly, we found that SPI1 is picked out in top three of priming macrophages and all states of microglia, and also important for primed and repressed macrophages (**Table S3**). SPI1 is known as a crucial transcription factor for the development of macrophage (31, 32). Our results suggested that the transcription activity of SPI1 is essential for macrophages and microglia maturation and polarization, leading to a tumor-harmful microenvironment formation.

**Figure 6:**
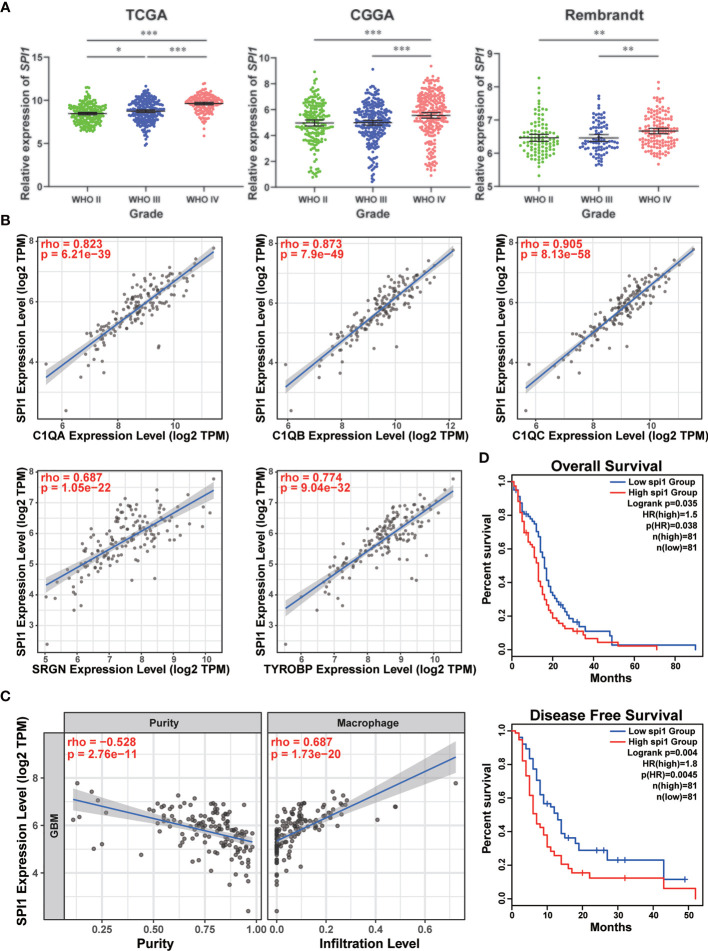
High expression of SPI1 correlated with upper macrophage infiltration and poor prognosis in GBM. **(A)** The expression levels of SPI1 in different WHO groups were calculated and visualized as scatter plots. *p < 0.05, **p < 0.01, ***p < 0.001. **(B)** The expression of SPI1 was positively correlated with marker genes in the TAM group. **(C)** The SPI1 expression was negatively correlated with tumor purity and positively correlated with macrophage infiltration. **(D)** The correlation between SPI1 expression and survival prognosis of GBM in TCGA. The OS and DFS maps were displayed.

In order to further clarify the vital role of SPI1 in GBM, the expression levels in different grades were measured by analyzing the transcriptome data in TCGA, CGGA, and Rembrandt databases. As shown in **Figure 6A**, grade IV (GBM) samples had the highest level of SPI1. As previously mentioned that marker genes of the TAM group included SPI1, C1QA, C1QB, C1QC, SRGN, and TYROBP (**Figure 1D**), we utilized Spearman’s rho value to interrogate the coexpression relationship between the expression level of SPI1 and these genes in GBM. After being analyzed in the TCGA-GBM dataset, the statistical positive correlations were observed between the SPI1 level and the genes expressed in the TAM group (C1QA: r = 0.823; C1QB: r = 0.873; C1QC: r = 0.905; SRGN: r = 0.687; TYROBP: r = 0.774) (**Figure 6B**), but not in the Tumor group (GAP43: r = 0.043; GPM6B: r = −0.135; SEC61G: r = −0.117; PTN: r = −0.298) (**Figure S8**). Meanwhile, we employed the TIMER algorithm to explore if there were potential relationships between the macrophage infiltration and the expression of these genes. We found that the elevated expression level of SPI1 had lower tumor purity and a higher infiltrating level of macrophage (Tumor purity: r = −0.528; macrophage infiltration: r = 0.687) (**Figure 6C**), so did TAM genes (**Figure S9A**). The tumor genes had no statistical correlation with tumor purity and macrophage infiltration (**Figure S9B**). Moreover, survival analysis using the Kaplan-Meier plotter tool displayed that the upregulated expression of SPI1 in tumor mass was linked to short overall survival (OS) and disease-free survival (DFS) time in patients with GBM (**Figure 6D**). Moreover, we inspected the likely downstream target genes of SPI1 in TAMs. By analyzing the glioma data in the TCGA database, the expression level of SPI1 displayed a significant positive correlation with target genes (CX3CR1: r = 0.53; CSF1R: r = 0.876; IRF8: r = 0.678) (**Figure S10A**). The co-expression signature was also observed in violin plots in single-cell data (**Figure S10B**).

The above results implied that tumor treatment targeting SPI1 could probably remodel the immune microenvironment of tumor and prolong the life span of GBM patients.

## Discussion

The tumor microenvironment (TME) is composed of an extracellular matrix interspersed with various cellular components, which regulates tumor progression *via* crosstalk and interplay with the tumor cells (33, 34). The TME is also associated with tumor metastasis and therapeutic resistance (35–38). Therefore, clarifying the specific characteristics of the TME in various cancers is particularly important in comprehensively understanding and theoretically targeting various tumors. GBM is a malignant neoplasm of the brain with a high heterogeneity and a poor prognosis. The exclusive nature of the brain tissue involved in GBM tumorigenesis determines the unique features of the microenvironment in this cancer (39). In recent years, the immune composition of GBM has emerged as a significant factor in multiple studies, suggesting that TAM populations account for a large number of cells within the tumor mass (19). By utilizing gene expression data from TCGA and GEO databases, Engler et al. discovered an enrichment in immune response–related gene signatures (particularly of TAM genes) in the mesenchymal subtype of GBM, suggesting that TAMs have a subtype-specific role in GBM (40). Another investigation leveraged a mouse model to demonstrate that the majority of GBM-associated macrophages are bone marrow–derived myeloid cells, which infiltrate the tumor during the early stages of oncogenesis and localize to the perivascular niche (41). The use of scRNA-seq to elucidate the gene signatures of *CD11b*-positive cells from GBM biopsies revealed that individual cells frequently coexpressed of both pro-inflammatory M1 and immune suppressive M2 macrophage genes (42), suggesting that the distinct partitioning of the M1/M2 macrophage subtypes is inadequate to completely represent the TAM expression profiles in GBM (43). However, to our knowledge, this is the first investigation to comprehensively describe the cell composition and transcriptomic characteristics of immune cells in GBM at the single-cell level.

**Figure 7:**
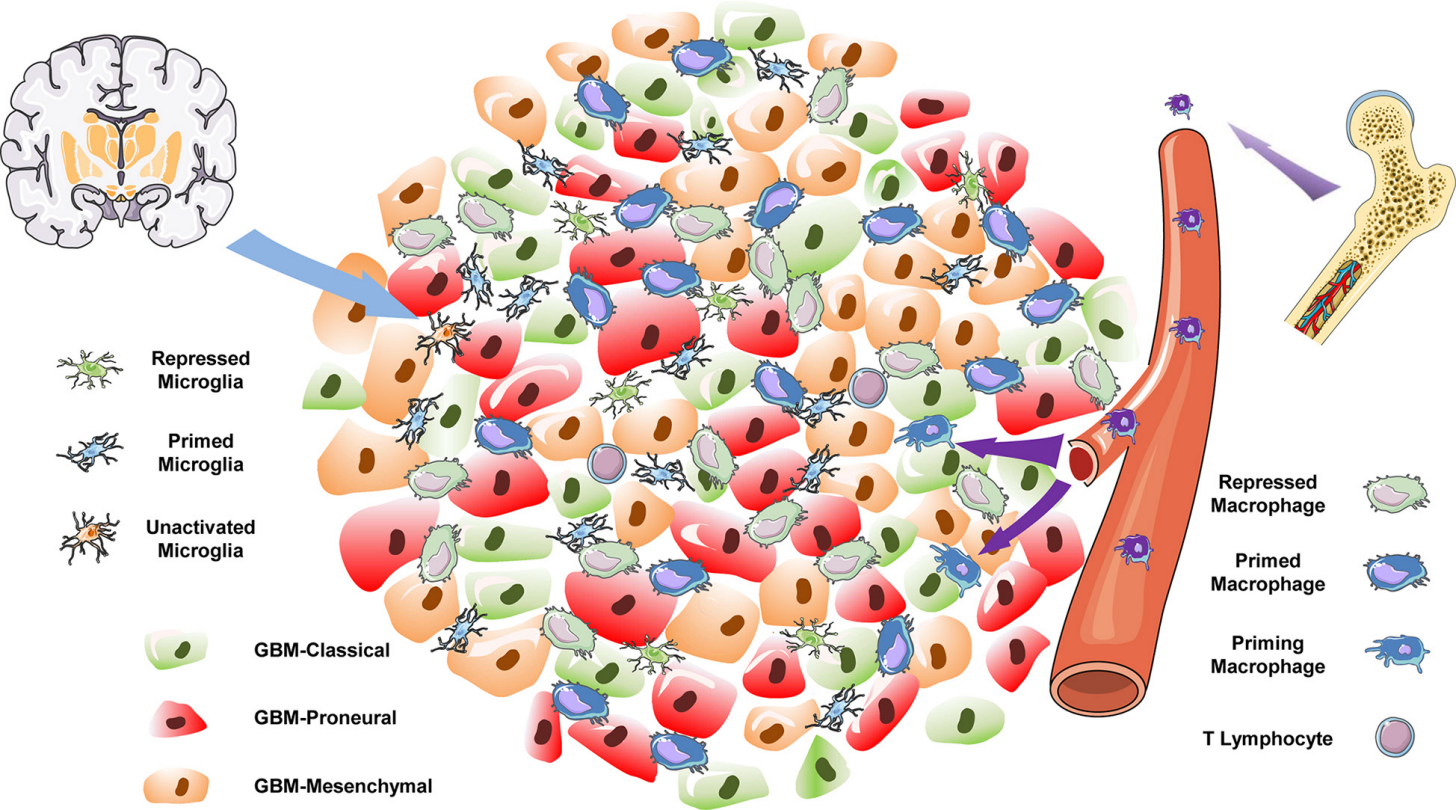
The immune landscape of GBM.

In the present study, we focused on the distinct origin and activation states of TAMs in different subtypes of GBM. In the TCGA-mixed tumor, TAMs were largely composed of primed (clusters 1 and 2) and repressed (cluster 0) macrophages (**Figure 3B**). In the classical subtype, repressed microglia occupied the main proportion of the total (**Figure 3C**). However, in the proneural GBM subtype, the proportions of repressed macrophages and primed microglia were similar, each comprising slightly more than 40% of the total cell population (**Figure 3D**). The classical and proneural subtypes shared TAM cells in the form of repressed macrophages (cluster 3) and priming macrophages (cluster 8). However, each GBM subtype also possessed unique TAMs: the primed microglia (cluster 4) were a prominent feature of the proneural subtype, whereas almost all repressed microglia (cluster 7) originated from the classical subtype. The KEGG pathway analysis also revealed differences in the main cellular biological processes of otherwise similar TAMs, such as the enrichment of “oxidative phosphorylation” pathways in cluster 0 of repressed macrophages but “protein processing in ER” in cluster 3. Unfortunately, the nine datasets analyzed for this study did not include a mesenchymal GBM subtype. However, it is worth emphasizing that GBM is notorious for its high heterogeneity and complex cell components, and different regions of the same tumor have been shown to exhibit features of the different subtypes, with distinct immune cell infiltration. The integrated analysis of the nine samples as a single large tumor mass may thus be a closer reflection of the natural status of GBM *in situ*. This integrated analysis has allowed us to depict the immune landscape of GBM at a single-cell level (**Figure 7**). It should be noted that the batch effect among these datasets still existed because the downloadable matrix is under mathematical manipulation and hard to restore. But in keeping with previous findings (19, 20), our results confirm that TAMs may occupy over one-third of the GBM tumor, with the ratio of the bone marrow–derived macrophages and the brain-resident microglia being 2:1, but infiltrating T lymphocytes comprising less than 2% of the tumor mass. Successful immunotherapy requires substantial infiltration of functional T cells within the tumors (44, 45). The limited number of T lymphocytes in GBM, which is isolated by the blood–brain barrier, means that immunotherapies targeting T cells such as monoclonal antibodies against programmed cell death 1 (PD-1) or cytotoxic T-lymphocyte-associated protein 4 (CTLA-4) are not suitable for treating GBM (46).

Given the large proportion of TAM-infiltrating GBM, the ability to convert immune-suppressed TAMs to a more proinflammatory state has become a promising immuno- therapeutic target. The colony-stimulating factor 1 receptor (CSF1R) plays essential roles in the development and function of macrophages and microglia (47). An inhibitor of the CSF1R was able to increase survival and decrease the tumor volume in a proneural GBM mouse model (48–50). In our study, we observed that *SPI1* was a vital regulon in all states of TAMs (**Figure 5** and **Table S3**). SPI1, also called PU.1, is a member of Ets family transcription factors and specifically expressed in lymphoid and myeloid cell lineages. The continuous high expression of SPI1 in hematopoietic cells results in macrophage differentiation (32). DB1976 is a furan-bisbenzimidazole-diamidine that strongly inhibits the transcription activity of SPI1 (51). We hypothesize that inhibition of SPI1 by utilizing DB1976 could reduce the TAM maturation and polarization, thus eliciting an antitumor effect.

## Conclusions

In summary, our comprehensive characterization of immune cells from a total of nine GBM tissues revealed a unique immune landscape in GBM at the single-cell level and identified SPI1 as a potential immunotherapeutic target against TAMs in GBM.

## Data Availability Statement

Publicly available datasets were analyzed in this study. This data can be found here: GSE131928.

## Author Contributions

Study conception and design: CK and XC. Acquisition of the nine scRNA-seq datasets: XC and FT. Dimensionality reduction and unsupervised clustering: XC, QW, and YW. Deep analysis (cell types and marker genes identification, gene regulatory network, KEGG pathway, etc.): XC, QW, JZ, and CX. Interpretation of data and manuscript writing: XC and CK. Study supervision: CK and HW. Funding acquisition: CK and XC. All authors have read and agreed the published version of the manuscript. 

## Funding

This research was supported by Beijing Tianjin Hebei Basic Research Cooperation Project (Nos. 18JCZDJC45500 and H2018201306), Tianjin Key R&D Plan of Tianjin Science and Technology Plan Project (No. 20YFZCSY00360), National Nature Science Foundation of China (No. 82002657), China Postdoctoral Science Foundation (No. 2020M670673), and Zhao Yi-Cheng Medical Science Foundation (No. ZYYFY2019002).

## Conflict of Interest

The authors declare that the research was conducted in the absence of any commercial or financial relationships that could be construed as a potential conflict of interest.

## Publisher’s Note

All claims expressed in this article are solely those of the authors and do not necessarily represent those of their affiliated organizations, or those of the publisher, the editors and the reviewers. Any product that may be evaluated in this article, or claim that may be made by its manufacturer, is not guaranteed or endorsed by the publisher.

## Supplementary Material

Supplementary Table 1 All differential expression genes for clusters.

Supplementary Table 2 All differential expression genes for macrophage clusters.

Supplementary Table 3 RSS of regulons in each cell type.

Supplementary Figure 1 Related to **Figure 1**. **(A)** The 22 clusters are identified and visualized as a t-SNE map. **(B)** The distribution of nine samples was displayed as a t-SNE map. **(C)** The expressions of CD3G, GZMH, IL2RB, PRF1, and ICOS distinguish T lymphocytes from the rest of the cell mass. **(D)** Two distinct aggregations are evident in the PCA plot.

Supplementary Figure 2 Related to **Figure 1**. **(A–I)** The cell types in each sample were identified and visualized as t-SNE plots. **(J)** The relative ratios of each cell type are displayed.

Supplementary Figure 3 Related to **Figure 2**. **(A)** A t-SNE plot showing the cell clusters. **(B–C)** The distribution of CD68 is visualized by a t-SNE plot and a violin plot. **(D)** Several genes (CYFIP1, ENTPD1, and VAV1) have similar expression signatures in comparison to TMEM119, indicating that these genes might be the marker genes of brain resident microglia. **(E)** The expression of MHC-II, in the form of HLA-DQA2 and HLA-DQB2, is restricted to cluster 1, 2, 4, 5, 6, 8, and 9. **(F)** The expression levels of TMEM119 and MHC-II divide these clusters into 4 main subtypes: TMEM119+-MHC-II+ cells, TMEM119+-MHC-II- cells, TMEM119--MHC-II+ cells, and TMEM119--MHC-II- cells.

Supplementary Figure 4 Related to **Figure 2**. **(A–B)** Different marker genes in microglia and macrophages. **(C)** The category schemes defining the cell types of TAM in GBM by the expression signatures of TMEM119 and MHC-II.

Supplementary Figure 5 Related to **Figure 3**. **(A–F)** The cell distributions of MGH102, MGH114, MGH118, MGH 125, MGH126 and MGH143 are displayed in t-SNE plots.

Supplementary Figure 6 Related to **Figure 5**. **(A)** Heat map display the relevancies among the regulons.

Supplementary Figure 7 Related to **Figure 5**. **(A–I)** t-SNE map showed the AUCell scores of crucial regulons (THAP1, ATF4, CREM, SPI1, JUND, REL, RUNX3, IRF7, STAT1).

Supplementary Figure 8 Related to **Figure 6**. The correlation between SPI1 expression and genes in tumor group.

Supplementary Figure 9 Related to **Figure 6**. **(A)** The expressions of TAM genes were negatively correlated with tumor purity and positively correlated with macrophage infiltration. **(B)** The expressions of tumor genes had no correlation with tumor purity and macrophage infiltration.

Supplementary Figure 10 Related to **Figure 6**. **(A)** The correlation between SPI1 expression and downstream target genes. **(B)** The distribution of SPI1 and downstream target genes are visualized by violin plots.
