# Supplementary figures and images for: Single-Cell Transcriptomics of Glioblastoma Reveals a Unique Tumor Microenvironment and Potential Immunotherapeutic Target Against Tumor-Associated Macrophage
Source: Front Oncol. 2021 Aug 9;11:710695. doi: 10.3389/fonc.2021.710695 (PMC8382282; doi:10.3389/fonc.2021.710695)

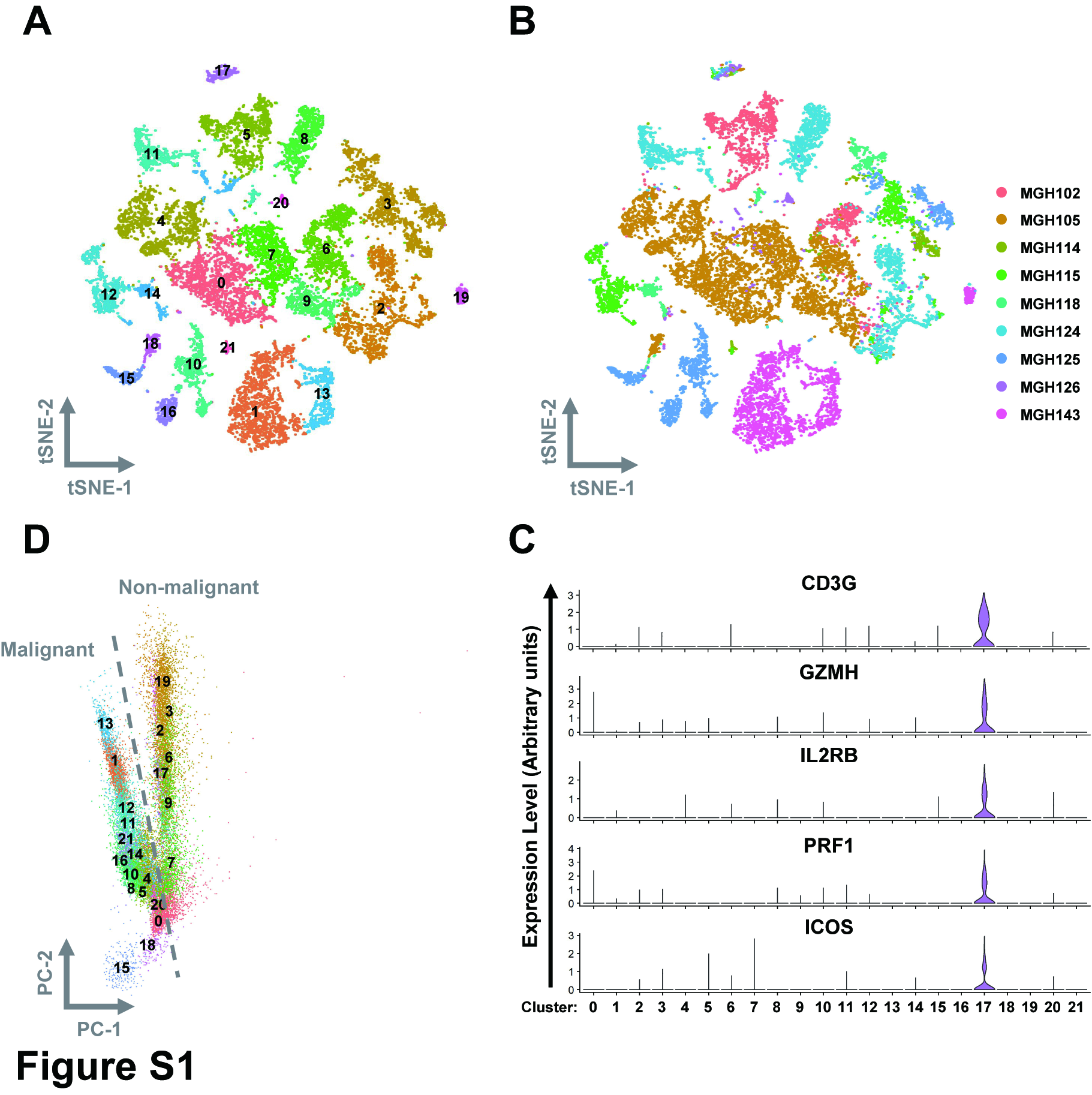

Supplement: Supplementary Figure 1 — Related to Figure 1. (A) The 22 clusters are identified and visualized as a t-SNE map. (B) The distribution of nine samples was displayed as a t-SNE map. (C) The expressions of CD3G, GZMH, IL2RB, PRF1, and ICOS distinguish T lymphocytes from the rest of the cell mass. (D) Two distinct aggregations are evident in the PCA plot. [file Image_1.tif]

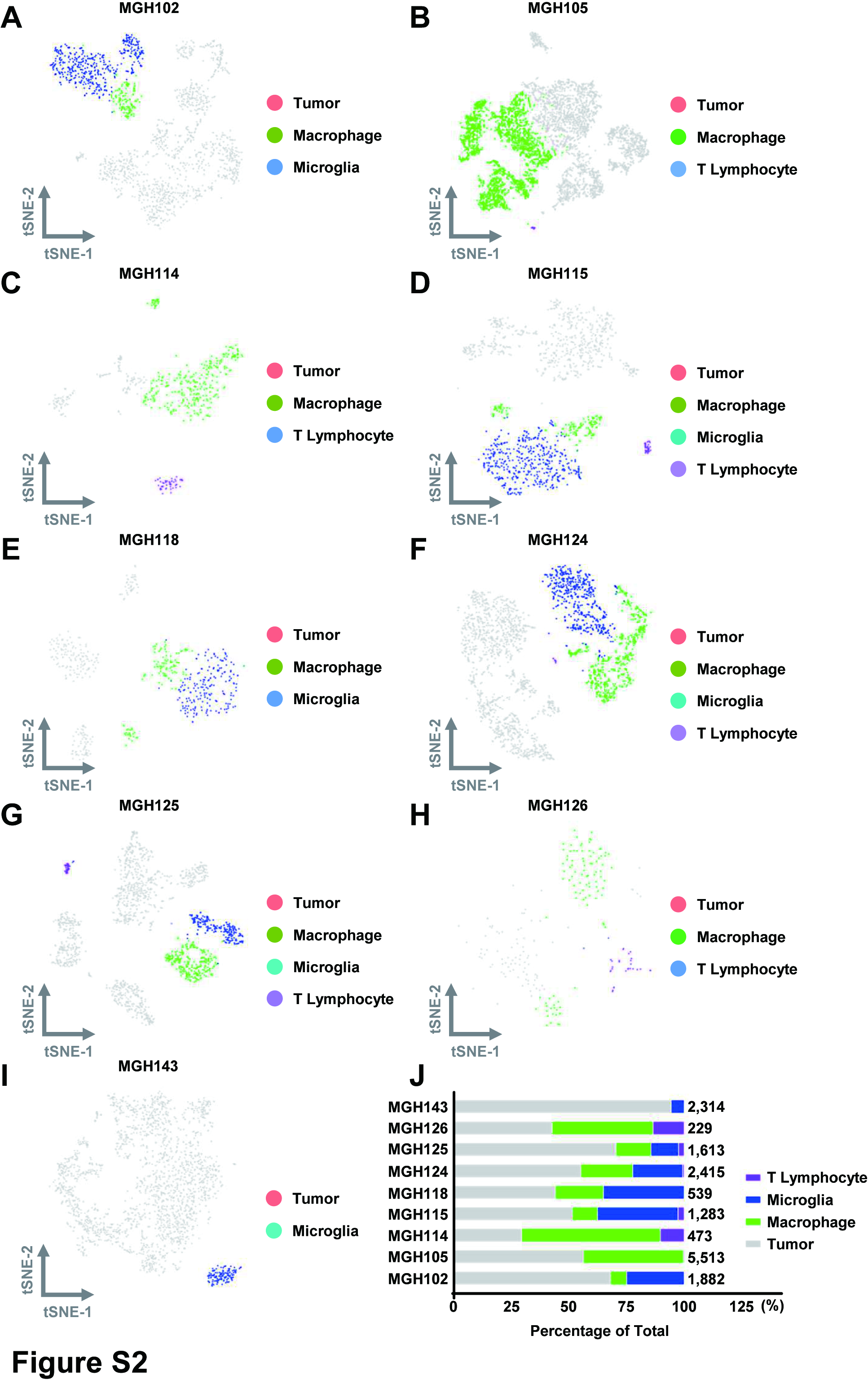

Supplement: Supplementary Figure 2 — Related to Figure 1. (A–I) The cell types in each sample were identified and visualized as t-SNE plots. (J) The relative ratios of each cell type are displayed. [file Image_2.tif]

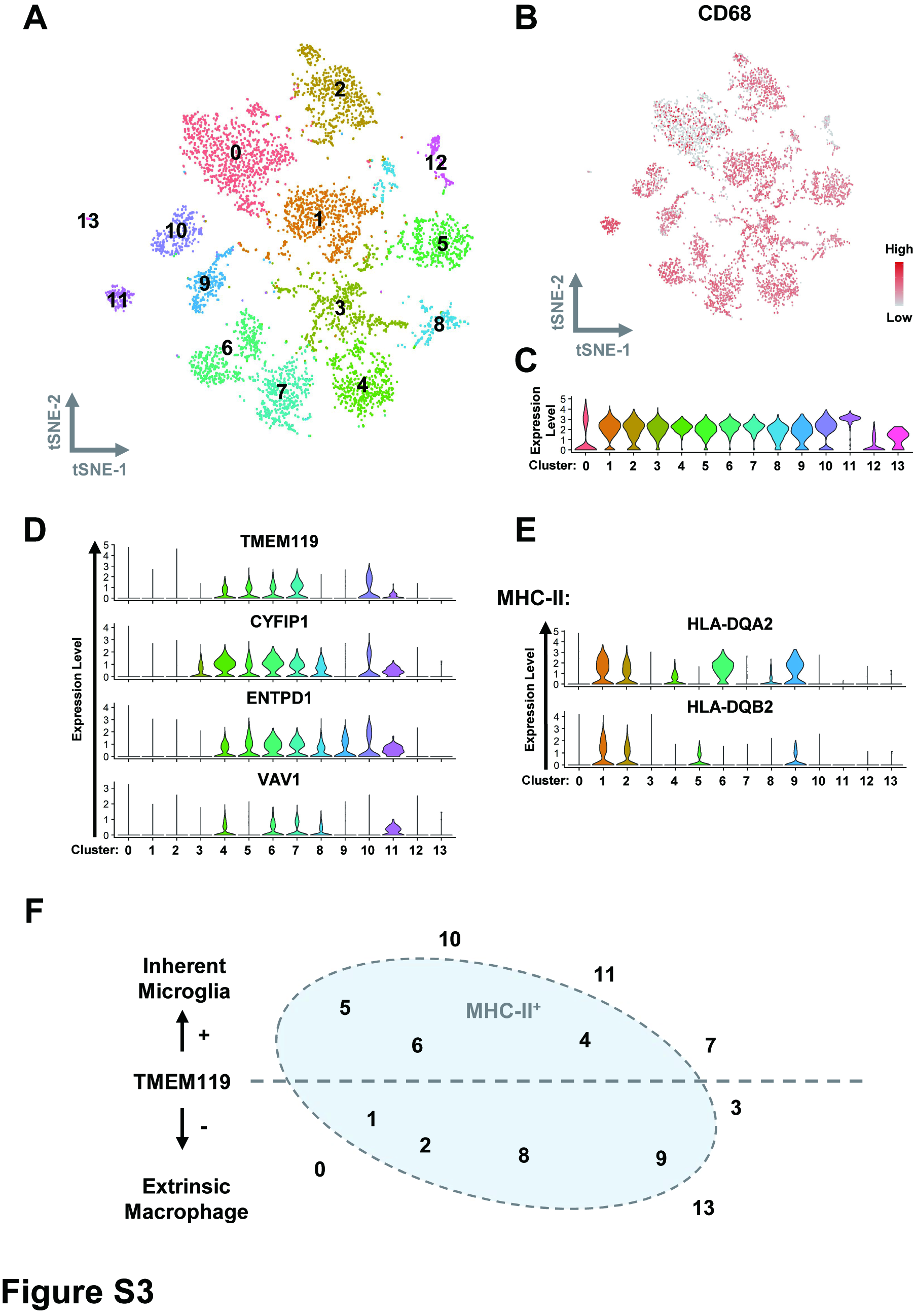

Supplement: Supplementary Figure 3 — Related to Figure 2. (A) A t-SNE plot showing the cell clusters. (B–C) The distribution of CD68 is visualized by a t-SNE plot and a violin plot. (D) Several genes (CYFIP1, ENTPD1, and VAV1) have similar expression signatures in comparison to TMEM119, indicating that these genes might be the marker genes of brain resident microglia. (E) The expression of MHC-II, in the form of HLA-DQA2 and HLA-DQB2, is restricted to cluster 1, 2, 4, 5, 6, 8, and 9. (F) The expression levels of TMEM119 and MHC-II divide these clusters into 4 main subtypes: TMEM119+-MHC-II+ cells, TMEM119+-MHC-II- cells, TMEM119--MHC-II+ cells, and TMEM119--MHC-II- cells. [file Image_3.tif]

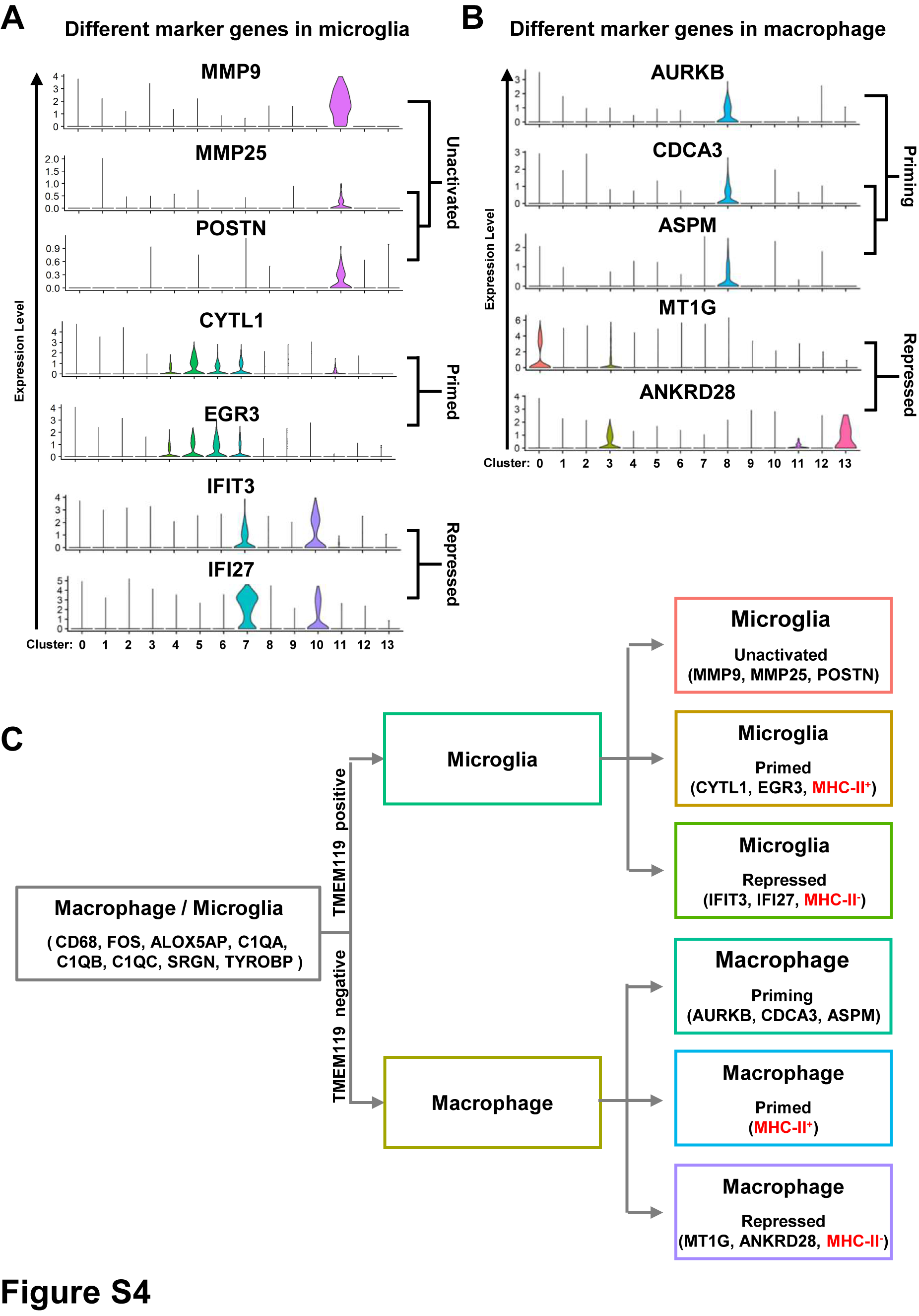

Supplement: Supplementary Figure 4 — Related to Figure 2. (A–B) Different marker genes in microglia and macrophages. (C) The category schemes defining the cell types of TAM in GBM by the expression signatures of TMEM119 and MHC-II. [file Image_4.tif]

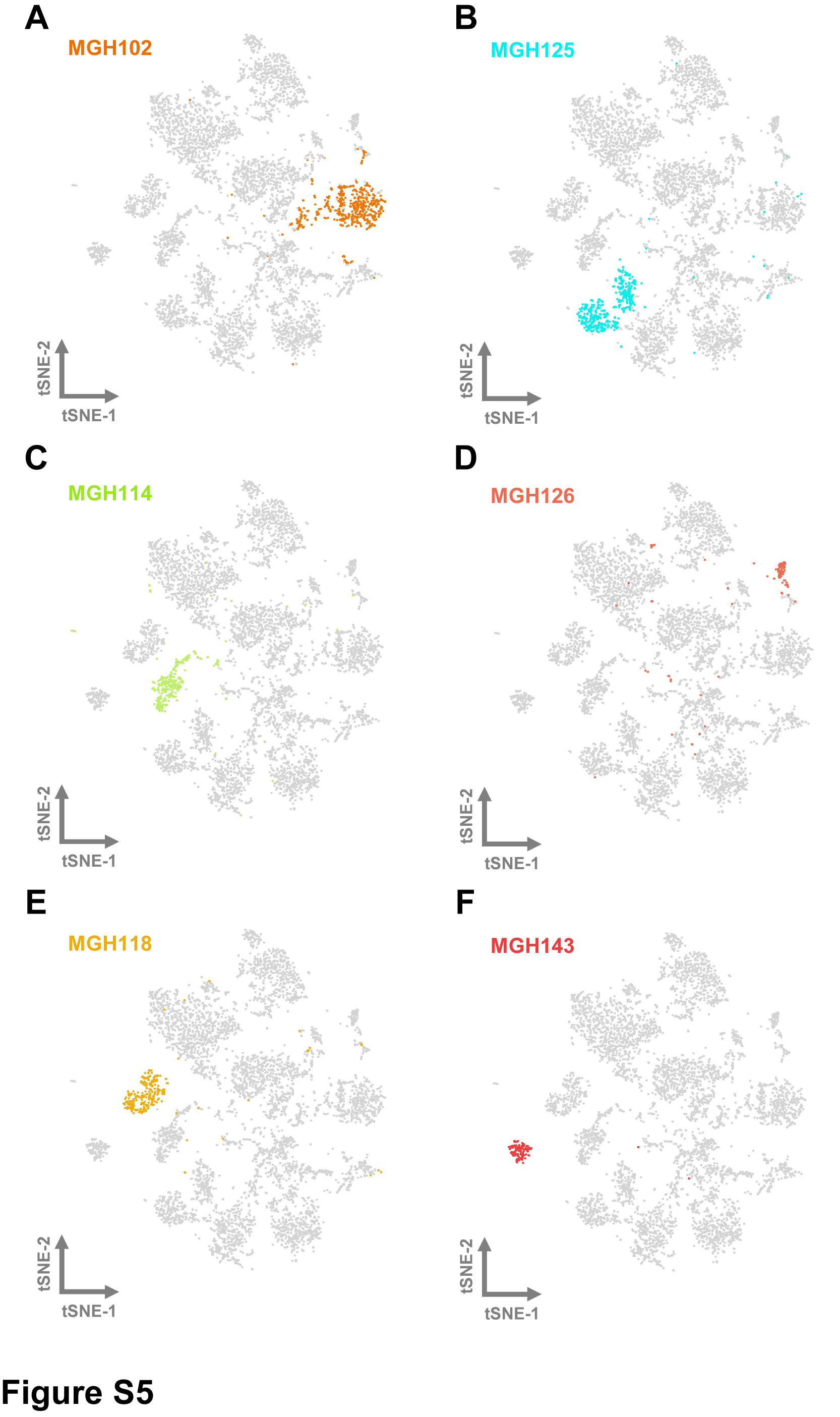

Supplement: Supplementary Figure 5 — Related to Figure 3. (A–F) The cell distributions of MGH102, MGH114, MGH118, MGH 125, MGH126 and MGH143 are displayed in t-SNE plots. [file Image_5.tif]

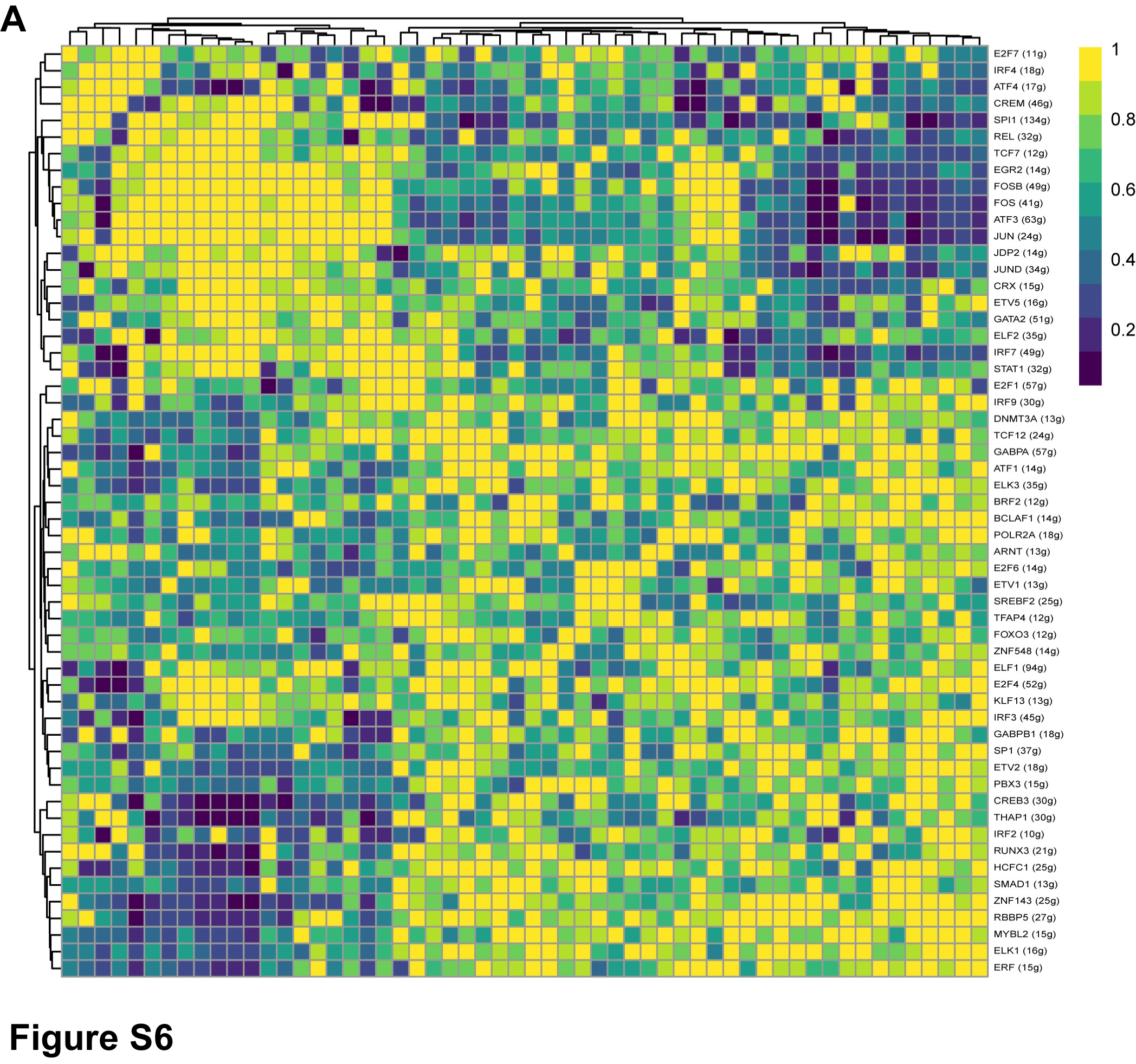

Supplement: Supplementary Figure 6 — Related to Figure 5. (A) Heat map display the relevancies among the regulons. [file Image_6.tif]

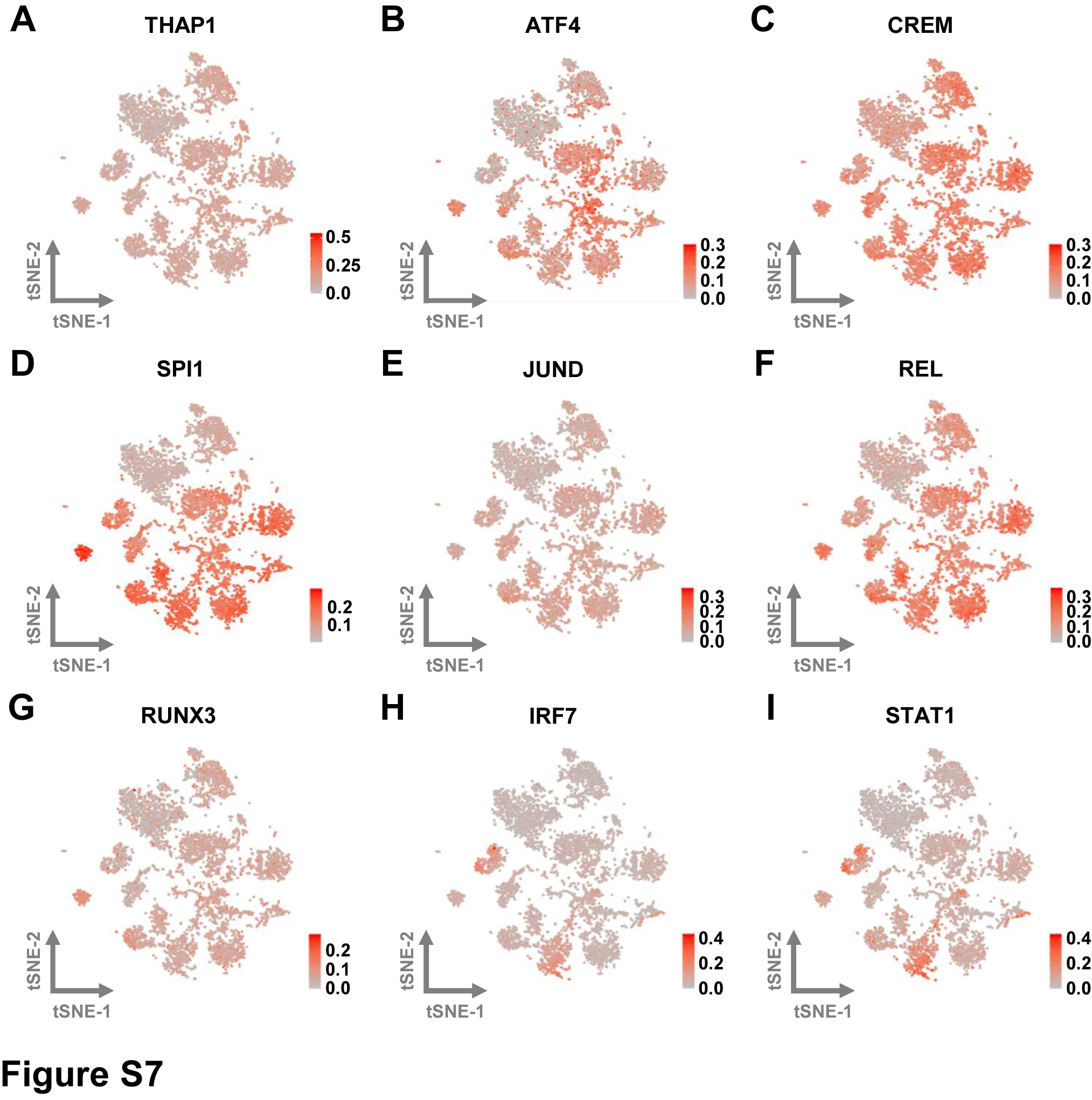

Supplement: Supplementary Figure 7 — Related to Figure 5. (A–I) t-SNE map showed the AUCell scores of crucial regulons (THAP1, ATF4, CREM, SPI1, JUND, REL, RUNX3, IRF7, STAT1). [file Image_7.tif]

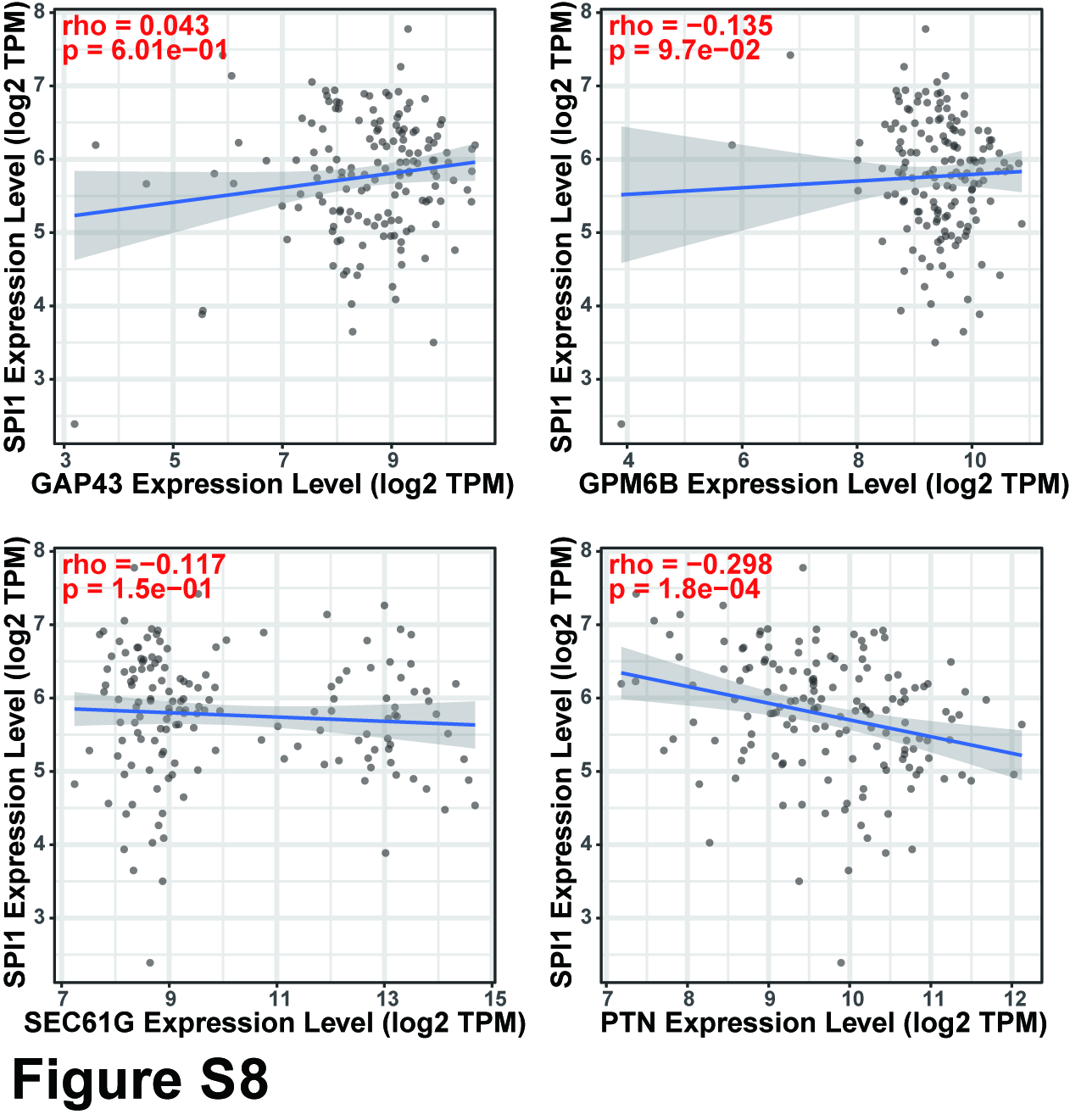

Supplement: Supplementary Figure 8 — Related to Figure 6. The correlation between SPI1 expression and genes in tumor group. [file Image_8.tif]

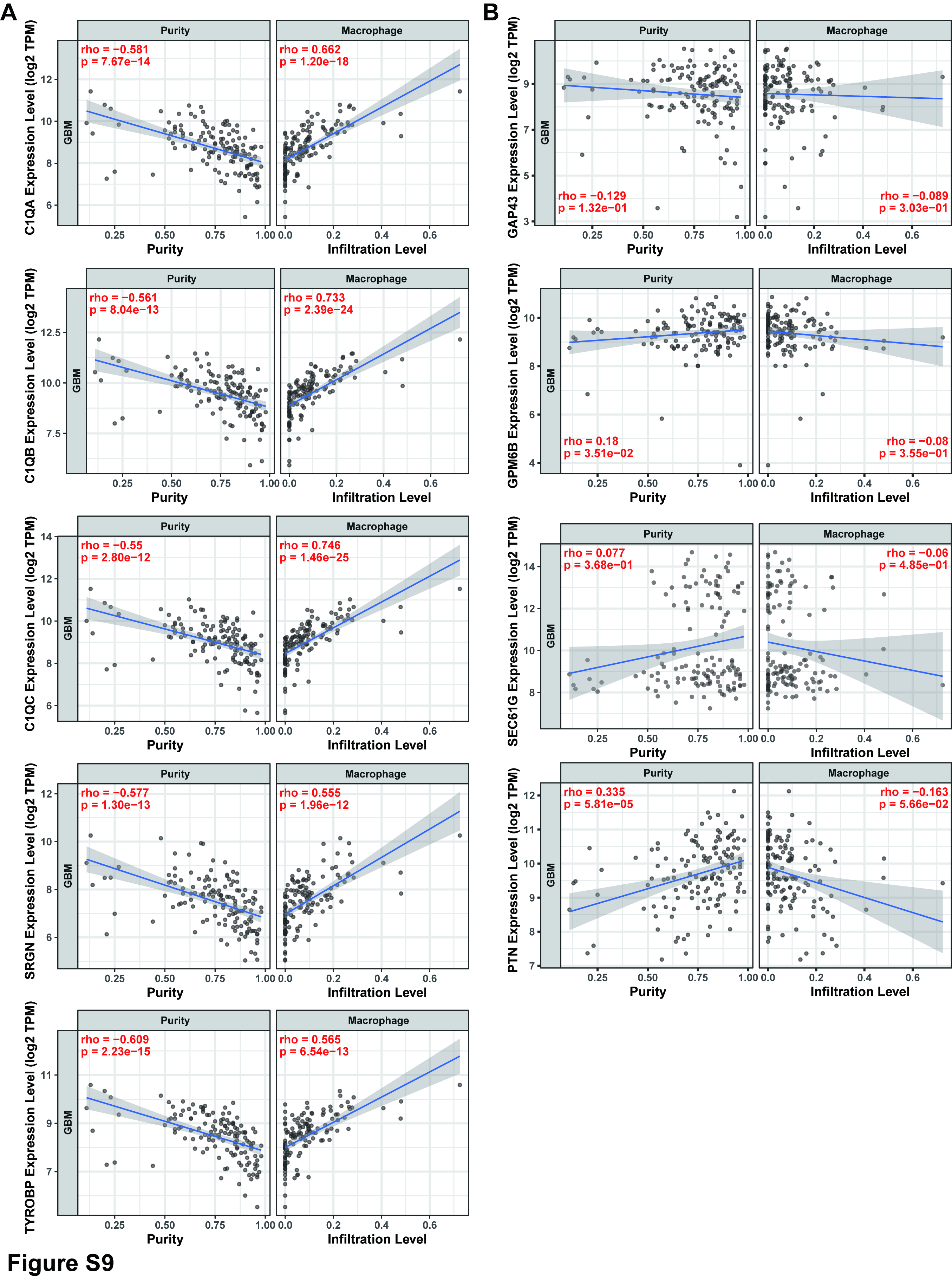

Supplement: Supplementary Figure 9 — Related to Figure 6. (A) The expressions of TAM genes were negatively correlated with tumor purity and positively correlated with macrophage infiltration. (B) The expressions of tumor genes had no correlation with tumor purity and macrophage infiltration. [file Image_9.tif]

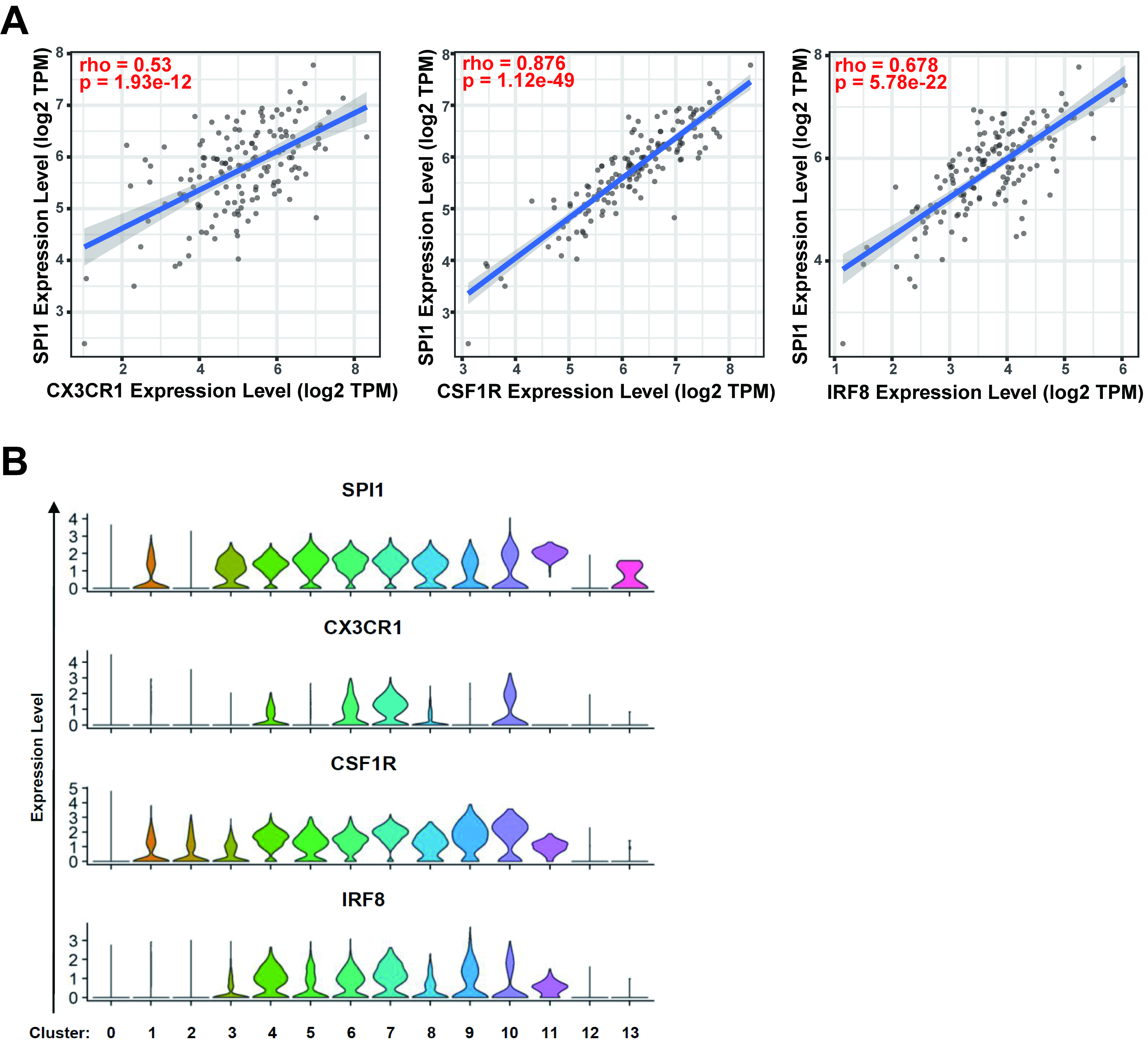

Supplement: Supplementary Figure 10 — Related to Figure 6. (A) The correlation between SPI1 expression and downstream target genes. (B) The distribution of SPI1 and downstream target genes are visualized by violin plots. [file Image_10.tif]
